# Global post‑marketing safety surveillance of Tumor Treating Fields (TTFields) therapy in over 25,000 patients with CNS malignancies treated between 2011–2022

**DOI:** 10.1007/s11060-024-04682-7

**Published:** 2024-06-29

**Authors:** Maciej M. Mrugala, Wenyin Shi, Fabio Iwomoto, Rimas V. Lukas, Joshua D. Palmer, John H. Suh, Martin Glas

**Affiliations:** 1 Mayo Clinic College of Medicine and Science, Mayo Clinic, Phoenix/Scottsdale, Arizona, USA; 2 Department of Radiation Oncology, Thomas Jefferson University, Philadelphia, PA USA; 3 Division of Neuro-Oncology, New York-Presbyterian/Columbia University Medical Center, New York, NY USA; 4 Department of Neurology, Northwestern University, Chicago, IL USA; 5 The Department of Radiation Oncology, The James Cancer Hospital, Ohio State University Wexner Medical Center, Columbus, OH USA; 6 Department of Radiation Oncology, Taussig Cancer Institute, Cleveland Clinic, Cleveland, OH USA; 7 Division of Clinical Neurooncology, Department of Neurology, University Hospital Essen, University Duisburg-Essen, West German Cancer Center (WTZ) and German Cancer Consortium, Partner Site, Essen, Germany

**Keywords:** Tumor Treating Fields, TTFields, Glioblastoma, Brain tumor, High grade gliomas, Safety

## Abstract

**Background:**

Tumor Treating Fields (TTFields) are alternating electric fields that disrupt cancer cell processes. TTFields therapy is approved for recurrent glioblastoma (rGBM), and newly-diagnosed (nd) GBM (with concomitant temozolomide for ndGBM; US), and for grade IV glioma (EU). We present an updated global, post-marketing surveillance safety analysis of patients with CNS malignancies treated with TTFields therapy.

**Methods:**

Safety data were collected from routine post-marketing activities for patients in North America, Europe, Israel, and Japan (October 2011–October 2022). Adverse events (AEs) were stratified by age, sex, and diagnosis.

**Results:**

Overall, 25,898 patients were included (diagnoses: ndGBM [68%], rGBM [26%], anaplastic astrocytoma/oligodendroglioma [4%], other CNS malignancies [2%]). Median (range) age was 59 (3–103) years; 66% patients were male. Most (69%) patients were 18–65 years; 0.4% were < 18 years; 30% were ﻿> 65 years. All-cause and TTFields-related AEs occurred in 18,798 (73%) and 14,599 (56%) patients, respectively. Most common treatment-related AEs were beneath-array skin reactions (43%), electric sensation (tingling; 14%), and heat sensation (warmth; 12%). Treatment-related skin reactions were comparable in pediatric (39%), adult (42%), and elderly (45%) groups, and in males (41%) and females (46%); and similar across diagnostic subgroups (ndGBM, 46%; rGBM, 34%; anaplastic astrocytoma/oligodendroglioma, 42%; other, 40%). No TTFields-related systemic AEs were reported.

**Conclusions:**

This long-term, real-world analysis of > 25,000 patients demonstrated good tolerability of TTFields in patients with CNS malignancies. Most therapy-related AEs were manageable localized, non-serious skin events. The TTFields therapy safety profile remained consistent across subgroups (age, sex, and diagnosis), indicative of its broad applicability.

**Supplementary Information:**

The online version contains supplementary material available at 10.1007/s11060-024-04682-7.

## Introduction

Malignant gliomas, including glioblastoma (GBM [grade 4 isocitrate dehydrogenase wildtype (IDHwt)]), high grade astrocytoma IDH-mutated, and high grade oligodendroglioma IDH-mutated and 1p/19q-codeleted, classified based on histological and molecular characteristics in accordance with current World Health Organization (WHO) 2021 classification [1], are one of the most common primary glial central nervous system (CNS) cancers worldwide [2]. GBM has a high recurrence rate and very poor prognosis [3–5].

Treatment of newly diagnosed GBM (ndGBM) according to the Stupp protocol; i.e., maximum safe resection, followed by radiotherapy with concomitant and maintenance temozolomide (TMZ), has shown a median overall survival (OS) of 14–16 months, and a low 5-year OS rate of 5–10% [6–9].

Tumor Treating Fields (TTFields) are electric fields that exert physical forces to disrupt cellular processes critical for cancer cell viability and tumor progression [10, 11]. TTFields therapy is locoregional and non-invasive; the fields are generated by a wearable medical device and delivered to the tumor via arrays placed on the scalp. TTFields therapy targets cancer cells through multiple mechanisms, and predominantly exert their effects through mitotic and motility disruption, downregulation of the DNA damage response, and enhancement of antitumor immunity [12–18]. Additionally, TTFields have effects on upregulation of autophagy, enhancement of cell membrane permeability, and weakening of the tight junctions that constitute the blood-brain barrier ﻿[19–22].

TTFields therapy is approved for use as monotherapy in recurrent GBM (rGBM), and with maintenance TMZ in ndGBM, and Conformité Européene (CE)-marked for grade IV glioma in the European Union (EU), based on results from the pivotal EF-11 and EF-14 studies, respectively [9, 17, 22–24]. TTFields therapy with maintenance TMZ is a category 1 recommended treatment in the NCCN Clinical Practice Guidelines in Oncology (NCCN Guidelines) for ndGBM [22, 24, 25].

In the EF-11 study (rGBM patients), while the primary objective (demonstration of superiority of TTFields vs comparator) was not met, efficacy of TTFields monotherapy was comparable with physician’s best choice treatment, with TTFields therapy also showing improvements in the safety profile and quality of life (QoL) [23]. In the EF-14 study (ndGBM patients), OS and progression- free survival (PFS) were extended by 4.9 months and 2.7 months, respectively, with TTFields therapy concomitant with TMZ versus TMZ alone, representing a significant improvement for both endpoints [9]. The benefit appeared sustained, with 5-year OS rates of 13% versus 5% in the TTFields therapy with TMZ and TMZ alone groups, respectively [9]. Furthermore, TTFields therapy did not have a negative influence on health-related QoL, except for a higher incidence of skin toxicity [26, 27]. TTFields therapy was not associated with an increase in systemic toxicity in either the EF-11 or EF-14 pivotal studies [9, 23]. Recent meta-analysis of comparative TTFields therapy studies also suggests survival is significantly improved with the addition of TTFields to systemic standard of care (SOC) in patients with ndGBM in the real-world setting (HR: 0.63; 95% CI 0.53–0.75; *p* < 0.001). Among post-approval studies, the pooled median OS was 22.6 months (95% CI 17.6–41.2) for patients treated with TTFields therapy and 17.4 months (95% CI 14.4–21.6) for those receiving standard radiochemotherapy only [28]. These data provide evidence of consistency of efficacy in the real world.

Besides GBM, TTFields therapy with pemetrexed plus platinum-based chemotherapy has also been approved in the US and Europe for patients with pleural mesothelioma, under the Humanitarian Device Exemption pathway, based on results from the STELLAR study [29–31]. In this study, TTFields therapy demonstrated encouraging OS results, together with a tolerable safety profile and no increase in systemic toxicity [30].

TTFields therapy given concurrently with SOC therapies is also in clinical development as a potential approach for several other solid tumor indications.

Unsolicited post-marketing surveillance (PMS) data from 11,029 patients with CNS tumors treated with TTFields therapy between October 2011 and February 2019 were previously reported, which confirmed the favorable safety profile demonstrated in clinical studies and registry/real-world data without any new safety concerns [4, 9, 23, 32]. A sub-analysis of this dataset confirmed that TTFields therapy also has a favorable safety profile in a high-risk patient population with GBM and hydrocephalus harboring programmable or non-programmable ventriculoperitoneal (VP) shunts [33]. Furthermore, analysis of pediatric data from the PMS dataset revealed no new safety signals in children or adolescents with CNS tumors [34].

The present global PMS safety analysis reports an update on the previously published surveillance data [4], in a cohort of more than 25,000 patients with CNS tumors treated with TTFields therapy over an 11-year period.

## Methods

This was a post-marketing surveillance analysis of patients with CNS tumors treated with TTFields therapy between October 2011 and October 2022 in North America, Europe, Israel, and Japan.

Unsolicited safety data were collected from routine post-marketing activities and interactions between the device manufacturer, patients, caregivers, and healthcare professionals (which included treating physicians, nurses, and other team members). Adverse events (AEs) were analyzed by the device manufacturer to determine seriousness and relatedness to TTFields therapy. AEs were classified using the Medical Dictionary for Regulatory Activities (MedDRA) version 25.1 and were graded as serious or non-serious. An AE was considered serious if it led to ≥1 of the following: (1) death; (2) life-threatening illness/injury; (3) permanent body structure/function impairment, including chronic disease; (4) in-patient hospitalization or prolongation of existing hospitalization; (5) medical/surgical intervention to prevent life-threatening illness, injury, or permanent body structure/function impairment; (6) fetal distress/death, congenital abnormality, or birth defect.

AEs were stratified by age (<18,18–65, and >65 years of age), sex, and diagnosis (ndGBM, rGBM, anaplastic astrocytoma and anaplastic oligodendroglioma, and other CNS tumors [including low-grade gliomas, high-grade gliomas, and metastases]). The historical diagnostic nomenclature was utilized as the majority of data collection occurred prior to the adoption of the WHO 2021 classification system.

Due to the retrospective nature of the analysis, AE occurrences were assessed via descriptive statistics.

## Results

### Baseline characteristics

A total of 25,898 patients were identified and included in this analysis. Post-marketing surveillance data for these patients were evaluated in the results. Baseline characteristics are summarized in Table 1. Most patients (n=18,665 [72%]) were from North America; 6,032 (23%) were from Europe or Israel, and 1,201 (5%) were from Japan. For patients with known age (n=25,718), median age (range) at start of treatment was 59 (3–103) years of age. The majority (17,817 [69%]) were 18–65 years of age; in total, there were 93 (0.4%) pediatric (<18 years of age) patients, and 7,808 (30%) elderly (>65 years of age) patients. Two-thirds (n=16,994 [66%]) of patients were male. Diagnoses were ndGBM (n=17,587 [68%]), rGBM (n=6,774 [26%]), anaplastic astrocytoma/oligodendroglioma (n=1,141 [4%]) and other (n=396 [2%]).

**Table 1:** Baseline characteristics of patients treated with TTFields therapy

Characteristic	Total	ndGBM	rGBM	AA/AO	Other^a^
n (%)	(N = 25,898)	(n = 17,587)	(n = 6,774)	(n = 1,141)	(n = 396)
Age (years of age)					
< 18	93 (< 1)	37 (< 1)	31 (< 1)	14 (1)	11 (3)
18–65	17,817 (69)	11,390 (65)	5,123 (76)	1,000 (88)	304 (77)
> 65	7,808 (30)	5,989 (34)	1,611 (24)	127 (11)	81 (20)
Unknown	180 (1)	171 (1)	9 (< 1)	N/A	N/A
Sex					
Male	16,994 (66)	11,427 (65)	4,530 (67)	780 (68)	257 (65)
Female	8,904 (34)	6,160 (35)	2,244 (33)	361 (32)	139 (35)
Region					
North America	18,665 (72)	12,042 (68)	5,402 (80)	869 (76)	352 (89)
Europe and Israel	6,032 (23)	4,347 (25)	1,369 (20)	272 (24)	44 (11)
Japan	1201 (5)	1198 (7)	3 (< 1)	N/A	N/A

AA, anaplastic astrocytoma; AO, anaplastic oligodendroglioma; NA, not applicable; ndGBM, newly diagnosed glioblastoma; rGBM, recurrent glioblastoma; TTFields, Tumor Treating Fields

^a^Includes high-grade gliomas, low-grade gliomas, and brain metastases

Percentages may not total to 100%, due to rounding

### Safety

Overall, 18,798 (73%) patients reported ≥1 (all-cause) AE, with the most common being skin reaction beneath the arrays (n=11,062 [43%] patients), electric sensation (i.e. under-array tingling; n=3,557 [14%]), and heat sensation (under-array warmth; n=3,083 [12%]) (Table 2). Electric sensation may be caused by displaced contact of the arrays on the scalp, potentially leading to arching across the gap between the arrays and scalp interface. Such electric sensations are not life-threatening electric shocks, but are commonly described as a tingling sensation beneath the arrays [4].

**Table 2:** Most common AEs, whether related to TTFields therapy or not, in patients treated with TTFields therapy by age, sex, and diagnosis, with an incidence of ≥ 5% in the total cohort

**MedDRA v25.1** **System Organ Class / preferred term** **n (%)**	**Total** **(N = 25,898)**	**Age (years of age)**			Sex		Diagnosis			
		** < 18** **(n=93)**	**18–65** **(n = 17,817)**	** > 65** **(n = 7,808)**	**Female** **(n = 8,904)**	**Male** **(n = 16,994)**	**ndGBM** **(n = 17,587)**	**rGBM** **(n = 6,774)**	**AA/AO** **(n = 1,141)**	**Other**^**a**^ (n = 396)
**Patients with ≥ 1 AE**	**18,798 (73)**	**58 (62)**	**12,795 (72)**	**5,849 (75)**	**6,710 (75)**	**12,090 (71)**	**13,201 (75)**	**4,515 (67)**	**796 (70)**	**286 (72)**
**Gastrointestinal disorders**	**1,467 (6)**	**8 (9)**	**1,003 (6)**	**452 (6)**	**571 (6)**	**896 (5)**	**1,068 (6)**	**322 (5)**	**54 (5)**	**23 (6)**
**General disorders and administration site conditions**	**9,486 (37)**	**26 (28)**	**6,650 (37)**	**2,773 (36)**	**3,523 (40)**	**5,963 (35)**	**6,689 (38)**	**2,269 (33)**	**403 (35)**	**125 (32)**
Electric sensation^b^	3,557 (14)	9 (10)	2,734 (15)	800 (10)	1,402 (16)	2,155 (13)	2,576 (15)	758 (11)	167 (15)	56 (14)
Fatigue/malaise	1,809 (7)	6 (6)	1,204 (7)	591 (8)	637 (7)	1,172 (7)	1300 (7)	420 (6)	69 (6)	20 (5)
General physical health deterioration	1,273 (5)	1 (1)	873 (5)	396 (5)	426 (5)	847 (5)	876 (5)	352 (5)	41 (4)	4 (1)
Heat sensation^c^	3,083 (12)	13 (14)	2,157 (12)	901 (12)	1,156 (13)	1,927 (11)	2,152 (12)	726 (11)	152 (13)	53 (13)
Pain	1,784 (7)	6 (6)	1,214 (7)	561 (7)	809 (9)	975 (6)	1,300 (7)	393 (6)	70 (6)	21 (5)
**Infections and infestations**	**1,984 (8)**	**5 (5)**	**1,238 (7)**	**735 (9)**	**739 (8)**	**1245 (7)**	**1,424 (8)**	**451 (7)**	**80 (7)**	**29 (7)**
**Injury, poisoning, and procedural complications**	**3,004 (12)**	**3 (3)**	**1,855 (10)**	**1,135 (15)**	**1,117 (13)**	**1,887 (11)**	**2,128 (12)**	**718 (11)**	**113 (10)**	**45 (11)**
Fall	1,995 (8)	3 (3)	1,129 (6)	861 (11)	713 (8)	1,282 (8)	1,367 (8)	520 (8)	77 (7)	31 (8)
**Musculoskeletal and connective tissue disorders**	**1,753 (7)**	**1 (1)**	**1,062 (6)**	**686 (9)**	**672 (8)**	**1,081 (6)**	**1,308 (7)**	**373 (6)**	**59 (5)**	**13 (3)**
Muscular weakness	1,247 (5)	1 (1)	708 (4)	534 (7)	457 (5)	790 (5)	910 (5)	290 (4)	38 (3)	9 (2)
**Nervous system disorders**	**8,510 (33)**	**28 (30)**	**5,859 (33)**	**2,585 (33)**	**3,028 (34)**	**5,482 (32)**	**5,878 (33)**	**2,171 (32)**	**323 (28)**	**138 (35)**
Cognitive disorder	1,201 (5)	-	702 (4)	492 (6)	345 (4)	856 (5)	857 (5)	290 (4)	36 (3)	18 (5)
Headache	2,446 (9)	12 (13)	1,878 (11)	549 (7)	967 (11)	1,479 (9)	1,649 (9)	646 (10)	103 (9)	48 (12)
Seizure	3,034 (12)	10 (11)	2,164 (12)	850 (11)	1049 (12)	1,985 (12)	2,032 (12)	845 (12)	117 (10)	40 (10)
**Psychiatric disorders**	**1,535 (6)**	**3 (3)**	**1,019 (6)**	**508 (7)**	**485 (5)**	**1,050 (6)**	**1,082 (6)**	**373 (6)**	**63 (6)**	**17 (4)**
**Skin and subcutaneous tissue disorders**	**11,436 (44)**	**36 (39)**	**7,732 (43)**	**3,614 (46)**	**4,221 (47)**	**7,215 (42)**	**8,387** **(48)**	**2,388 (35)**	**499 (44)**	**162 (41)**
Skin reaction	11,062 (43)	36 (39)	7,444 (42)	3,528 (45)	4,123 (46)	6,939 (41)	8,143 (46)	2,279 (34)	483 (42)	157 (40)

AA, anaplastic astrocytoma; AE, adverse event; AO, anaplastic oligodendroglioma; MedDRA, Medical Dictionary for Regulatory Activities; ndGBM, newly diagnosed glioblastoma; rGBM, recurrent glioblastoma; TTFields, Tumor Treating Fields

^a^Includes high-grade gliomas, low-grade gliomas, and brain metastases; ^b^Commonly described as a tingling sensation below the arrays; ^c^heat below the arrays; commonly described as a warm sensation

Percentages may not total to 100%, due to rounding

AE patterns were generally similar regardless of age, sex, and diagnosis, although the incidence of skin reactions was slightly higher in patients with ndGBM than those with rGBM (n=8,143 [46%] vs n=2,279 [34%] patients, respectively) (Table 2).

In total, 58 (62%) of pediatric patients reported ≥1 AE, regardless of relatedness of TTFields therapy; the most common events were skin reaction (36 [39%]) patients, heat sensation (13 [14%]), and headache (12 [13%]), which were generally in line with the overall population.

Overall, 5,849 (75%) of elderly patients reported ≥1 AE. AEs in elderly patients were also consistent with the overall population; most common events were skin reaction (n=3,528 [45%] patients), heat sensation (n=901 [12%]), and electric sensation (n=800 [10%]).

**Fig. 1:**
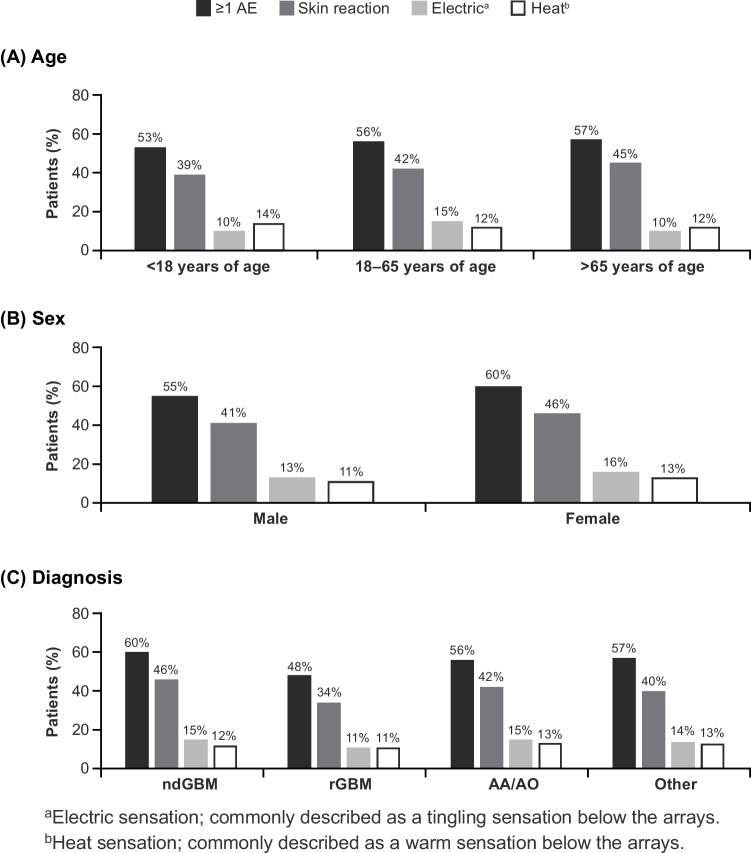
TTFields therapy-related AEs occurring in ≥ 10% of patients in any group, by age, sex, and diagnosis

In total, 14,599 (56%) of patients reported AEs considered by the manufacturer to be possibly related to TTFields therapy. Device-related AEs occurring in ≥2% of the overall population are shown in Supplementary Table 1. The most frequently reported device-related AEs were skin reaction (n=11,029 [43%] patients), electric sensation (n=3,557 [14%]) and heat sensation (n=3,083 [12%]), occurring mainly underneath the arrays (Figure 1). Other device-related AEs included skin ulcer and wound complication (each 1% [Table 3]).

**Table 3:** Most common TTFields therapy-related AEs by age, sex, and diagnosis: full data set

MedDRA v25.1 System Organ Class / preferred term n (%)	Total (N = 25,898)	Age (years)			Sex		Diagnosis			
		< 18 (n = 93)	18–65 (n = 17,817)	> 65 (n = 7,808)	Female (n = 8,904)	Male (n = 16,994)	ndGBM (n = 17,587)	rGBM (n = 6,774)	AA/AO (n = 1,141)	Other^a^ (n = 396)
**Ear and labyrinth disorders**	**5 (< 1)**	**-**	**3 (< 1)**	**2 (< 1)**	**2 (< 1)**	**3 (< 1)**	**4 (< 1)**	**-**	**1 (< 1)**	**-**
Auditory disorder	4 (< 1)	-	3 (< 1)	1 (< 1)	2 (< 1)	2 (< 1)	3 (< 1)	-	1 (< 1)	-
Ear disorder	1 (< 1)	-	-	1 (< 1)	-	1 (< 1)	1 (< 1)	-	-	-
**Eye disorders**	**9 (< 1)**	**-**	**7 (< 1)**	**2 (< 1)**	**1 (< 1)**	**8 (< 1)**	**8 (< 1)**	**1 (< 1)**	**-**	**-**
Eye disorder	5 (< 1)	-	4 (< 1)	1 (< 1)	1 (< 1)	4 (< 1)	5 (< 1)	-	-	-
Visual impairment	4 (< 1)	-	3 (< 1)	1 (< 1)	-	4 (< 1)	3 (< 1)	1 (< 1)	-	-
**Gastrointestinal disorders**	**19 (< 1)**	**-**	**16 (< 1)**	**3 (< 1)**	**6 (< 1)**	**13 (< 1)**	**16 (< 1)**	**2 (< 1)**	**-**	**1 (< 1)**
Nausea/vomiting	19 (< 1)	-	16 (< 1)	3 (< 1)	6 (< 1)	13 (< 1)	16 (< 1)	2 (< 1)	-	1 (< 1)
**General disorders and administration site conditions**	**7,356 (28)**	**22 (24)**	**5,250 (29)**	**2,056 (26)**	**2,797 (31)**	**4,559 (27)**	**5,232 (30)**	**1,675 (25)**	**337 (30)**	**112 (28)**
Complication associated with device	17 (< 1)	-	8 (< 1)	9 (< 1)	4 (< 1)	13 (< 1)	14 (< 1)	2 (< 1)	1 (< 1)	-
Discomfort	609 (2)	2 (2)	414 (2)	192 (2)	239 (3)	370 (2)	458 (3)	113 (2)	29 (3)	9 (2)
Electric sensation^b^	3,557 (14)	9 (10)	2,734 (15)	800 (10)	1,402 (16)	2,155 (13)	2,576 (15)	758 (11)	167 (15)	56 (14)
Fatigue/malaise	1,337 (5)	5 (5)	871 (5)	455 (6)	461 (5)	876 (5)	982 (6)	287 (4)	52 (5)	16 (4)
Gait disturbance	25 (< 1)	-	18 (< 1)	7 (< 1)	13 (< 1)	12 (< 1)	19 (< 1)	6 (< 1)	-	-
Heat sensation^c^	3,083 (12)	13 (14)	2157 (12)	901 (12)	1,156 (13)	1,927 (11)	2,152 (12)	726 (11)	152 (13)	53 (13)
Medical device site reaction	1 (< 1)	-	1 (< 1)	-	1 (< 1)	-	1 (< 1)	-	-	-
Oedema	16 (< 1)	-	12 (< 1)	4 (< 1)	9 (< 1)	7 (< 1)	16 (< 1)	-	-	-
Pain	1,227 (5)	3 (3)	852 (5)	371 (5)	566 (6)	661 (4)	913 (5)	251 (4)	51 (4)	12 (3)
Pyrexia	1 (< 1)	-	1 (< 1)	-	1 (< 1)	-	-	1 (< 1)	-	-
Swelling	1 (< 1)	-	1 (< 1)	-	1 (< 1)	-	1 (< 1)	-	-	-
Temperature intolerance	24 (< 1)	-	20 (< 1)	4 (< 1)	11 (< 1)	13 (< 1)	19 (< 1)	3 (< 1)	1 (< 1)	1 (< 1)
**Immune system disorders**	**84 (< 1)**	**-**	**52 (< 1)**	**32 (< 1)**	**27 (< 1)**	**57 (< 1)**	**74 (< 1)**	**7 (< 1)**	**3 (< 1)**	**-**
Hypersensitivity	84 (< 1)	-	52 (< 1)	32 (< 1)	27 (< 1)	57 (< 1)	74 (< 1)	7 (< 1)	3 (< 1)	-
**Infections and infestations**	**54 (< 1)**	**-**	**40 (< 1)**	**14 (< 1)**	**17 (< 1)**	**37 (< 1)**	**44 (< 1)**	**5 (< 1)**	**3 (< 1)**	**2 (1)**
Abscess	6 (< 1)	-	5 (< 1)	1 (< 1)	2 (< 1)	4 (< 1)	5 (< 1)	1 (< 1)	-	-
Brain abscess	1 (< 1)	-	1 (< 1)	-	1 (< 1)	-	1 (< 1)	-	-	-
Cellulitis	2 (< 1)	-	1 (< 1)	1 (< 1)	1 (< 1)	1 (< 1)	1 (< 1)	1 (< 1)	-	-
Eye infection	2 (< 1)	-	1 (< 1)	1 (< 1)	1 (< 1)	1 (< 1)	2 (< 1)	-	-	-
Infection	4 (< 1)	-	2 (< 1)	2 (< 1)	2 (< 1)	2 (< 1)	4 (< 1)	-	-	-
Osteomyelitis	1 (< 1)	-	1 (< 1)	-	-	1 (< 1)	1 (< 1)	-	-	-
Skin infection	38 (< 1)	-	29 (< 1)	9 (< 1)	10 (< 1)	28 (< 1)	30 (< 1)	3 (< 1)	3 (< 1)	2 (1)
**Injury, poisoning and procedural complications**	**496 (2)**	**-**	**323 (2)**	**167 (2)**	**234 (3)**	**262 (2)**	**365 (2)**	**109 (2)**	**16 (1)**	**6 (2)**
Contusion	36 (< 1)	-	23 (< 1)	13 (< 1)	16 (< 1)	20 (< 1)	28 (< 1)	8 (< 1)	-	-
Fall	114 (< 1)	-	72 (< 1)	42 (1)	44 (< 1)	70 (< 1)	69 (< 1)	38 (1)	6 (1)	1 (< 1)
Fracture	9 (< 1)	-	6 (< 1)	3 (< 1)	7 (< 1)	2 (< 1)	9 (< 1)	-	-	-
Injury	28 (< 1)	-	14 (< 1)	14 (< 1)	14 (< 1)	14 (< 1)	24 (< 1)	3 (< 1)	1 (< 1)	-
Radiation injury	4 (< 1)	-	1 (< 1)	3 (< 1)	2 (< 1)	2 (< 1)	3 (< 1)	1 (< 1)	(< 1)	-
Skin laceration	79 (< 1)	-	44 (< 1)	35 (< 1)	29 (< 1)	50 (< 1)	54 (< 1)	23 (< 1)	2 (< 1)	-
Thermal burn	9 (< 1)	-	6 (< 1)	3 (< 1)	6 (< 1)	3 (< 1)	7 (< 1)	2 (< 1)	-	-
Wound complication	257 (1)	-	183 (1)	68 (1)	137 (2)	120 (1)	202 (1)	43 (1)	7 (1)	5 (1)
**Investigations**	**405 (2)**	**-**	**280 (2)**	**124 (2)**	**149 (2)**	**256 (2)**	**328 (2)**	**59 (1)**	**13 (1)**	**5 (1)**
Quality of life decreased	405 (2)	-	280 (2)	124 (2)	149 (2)	256 (2)	328 (2)	59 (1)	13 (1)	5 (1)
**Metabolism and nutrition disorders**	**4 (< 1)**	**-**	**2 (< 1)**	**2 (< 1)**	**2 (< 1)**	**2 (< 1)**	**4 (< 1)**	**-**	**-**	**-**
Appetite disorder	4 (< 1)	-	2 (< 1)	2 (< 1)	2 (< 1)	2 (< 1)	4 (< 1)	-	-	-
**Musculoskeletal and connective tissue disorders**	**172 (1)**	**-**	**122 (1)**	**50 (1)**	**88 (1)**	**84 (< 1)**	**133 (1)**	**31 (< 1)**	**6 (1)**	**2 (1)**
Arthralgia	64 (< 1)	-	47 (< 1)	17 (< 1)	38 (< 1)	26 (< 1)	56 (< 1)	7 (< 1)	1 (< 1)	-
Arthritis	1 (< 1)	-	1 (< 1)	-	1 (< 1)	-	1 (< 1)	-	-	-
Arthropathy	1 (< 1)	-	-	1 (< 1)	-	1 (< 1)	1 (< 1)	-	-	-
Back disorder	1 (< 1)	-	1 (< 1)	-	1 (< 1)	-	1 (< 1)	-	-	-
Mobility decreased	6 (< 1)	-	2 (< 1)	4 (< 1)	1 (< 1)	5 (< 1)	5 (< 1)	1 (< 1)	-	-
Muscle spasms	60 (< 1)	-	44 (< 1)	16 (< 1)	25 (< 1)	35 (< 1)	38 (< 1)	16 (< 1)	5 (< 1)	1 (< 1)
Muscular weakness	14 (< 1)	-	8 (< 1)	6 (< 1)	5 (< 1)	9 (< 1)	8 (< 1)	5 (< 1)	-	1 (< 1)
Musculoskeletal stiffness	28 (< 1)	-	22 (< 1)	6 (< 1)	20 (< 1)	8 (< 1)	26 (< 1)	2 (< 1)	-	-
**Nervous system disorders**	**2,251 (9)**	**11 (12)**	**1,708 (10)**	**526 (7)**	**891 (10)**	**1,360 (8)**	**1,544 (9)**	**572 (8)**	**94 (8)**	**41 (10)**
Balance disorder	87 (< 1)	-	55 (< 1)	32 (< 1)	43 (< 1)	44 (< 1)	67 (< 1)	16 (< 1)	3 (< 1)	1 (< 1)
Cognitive disorder	19 (< 1)	-	14 (< 1)	5 (< 1)	4 (< 1)	15 (< 1)	16 (< 1)	3 (< 1)	-	-
Coordination abnormal	2 (< 1)	-	2 (< 1)	-	1 (< 1)	1 (< 1)	1 (< 1)	1 (< 1)	-	-
Dizziness	8 (< 1)	-	5 (< 1)	3 (< 1)	3 (< 1)	5 (< 1)	7 (< 1)	1 (< 1)	-	-
Dysesthesia	13 (< 1)	-	11 (< 1)	2 (< 1)	6 (< 1)	7 (< 1)	5 (< 1)	7 (< 1)	1 (< 1)	-
Headache	2,144 (8)	11 (12)	1,639 (9)	488 (6)	845 (9)	1,299 (8)	1,463 (8)	550 (8)	91 (8)	40 (10)
Hyperesthesia	1 (< 1)	-	1 (< 1)	-	-	1 (< 1)	-	-	1 (< 1)	-
Hypoesthesia	1 (< 1)	-	-	1 (< 1)	-	1 (< 1)	1 (< 1)	-	-	-
Lethargy	2 (< 1)	-	1 (< 1)	1 (< 1)	1 (< 1)	1 (< 1)	2 (< 1)	-	-	-
Memory impairment	2 (< 1)	-	1 (< 1)	1 (< 1)	-	2 (< 1)	2 (< 1)	-	-	-
Paresthesia	5 (< 1)	-	5 (< 1)	-	3 (< 1)	2 (< 1)	5 (< 1)	-	-	-
Speech disorder	7 (< 1)	-	5 (< 1)	2 (< 1)	1 (< 1)	6 (< 1)	7 (< 1)	-	-	-
Syncope	1 (< 1)	-	1 (< 1)	-	-	1 (< 1)	-	1 (< 1)	-	-
Tremor	1 (< 1)	-	-	1 (< 1)	1 (< 1)	-	1 (< 1)	-	-	-
**Psychiatric disorders**	**353 (1)**	**-**	**239 (1)**	**113 (1)**	**113 (1)**	**240 (1)**	**269 (2)**	**63 (1)**	**16 (1)**	**5 (1)**
Agitation	29 (< 1)	-	19 (< 1)	10 (< 1)	5 (< 1)	24 (< 1)	17 (< 1)	7 (< 1)	4 (< 1)	1 (< 1)
Anxiety	49 (< 1)	-	29 (< 1)	20 (< 1)	15 (< 1)	34 (< 1)	38 (< 1)	8 (< 1)	2 (< 1)	1 (< 1)
Claustrophobia	12 (< 1)	-	8 (< 1)	4 (< 1)	5 (< 1)	7 (< 1)	9 (< 1)	2 (< 1)	1 (< 1)	-
Depression	32 (< 1)	-	23 (< 1)	9 (< 1)	15 (< 1)	17 (< 1)	21 (< 1)	6 (< 1)	4 (< 1)	1 (< 1)
Insomnia	237 (1)	-	161 (1)	75 (1)	78 (1)	159 (1)	189 (1)	40 (1)	6 (1)	2 (1)
Mental status changes	2 (< 1)	-	1 (< 1)	1 (< 1)	-	2 (< 1)	1 (< 1)	1 (< 1)	-	-
Mood altered	2 (< 1)	-	2 (< 1)	-	1 (< 1)	1 (< 1)	2 (< 1)	-	-	-
Sleep disorder	2 (< 1)	-	2 (< 1)	-	-	2 (< 1)	1 (< 1)	1 (< 1)	-	-
Stress	15 (< 1)	-	9 (< 1)	6 (< 1)	3 (< 1)	12 (< 1)	13 (< 1)	2 (< 1)	-	-
**Respiratory, thoracic, and mediastinal disorders**	**4 (< 1)**	**-**	**4 (< 1)**	**-**	**1 (< 1)**	**3 (< 1)**	**4 (< 1)**	**-**	**-**	**-**
Asphyxia	4 (< 1)	-	4 (< 1)	-	1 (< 1)	3 (< 1)	4 (< 1)	-	-	-
**Skin and subcutaneous tissue disorders**	**11,193 (43)**	**36 (39)**	**7,550 (42)**	**3,554 (46)**	**4,147 (47)**	**7,046 (41)**	**8,228 (47)**	**2,314 (34)**	**493 (43)**	**158 (40)**
Alopecia	25 (< 1)	1 (1)	21 (< 1)	3 (< 1)	15 (< 1)	10 (< 1)	24 (< 1)	-	1 (< 1)	-
Decubitus ulcer	2 (< 1)	1 (1)	-	1 (< 1)	2 (< 1)	-	1 (< 1)	-	1 (< 1)	-
Hyperhidrosis	394 (2)	1 (1)	311 (2)	81 (1)	95 (1)	299 (2)	296 (2)	74 (1)	19 (2)	5 (1)
Purpura	1 (< 1)	-	1 (< 1)	-	1 (< 1)	-	-	-	1 (< 1)	-
Rash	11 (< 1)	-	6 (< 1)	5 (< 1)	3 (< 1)	8 (< 1)	9 (< 1)	2 (< 1)	-	-
Skin atrophy	5 (< 1)	-	4 (< 1)	1 (< 1)	3 (< 1)	2 (< 1)	4 (< 1)	-	1 (< 1)	-
Skin discoloration	5 (< 1)	-	5 (< 1)	-	1 (< 1)	4 (< 1)	4 (< 1)	1 (< 1)	-	-
Skin erosion	11 (< 1)	-	9 (< 1)	2 (< 1)	7 (< 1)	4 (< 1)	8 (< 1)	1 (< 1)	2 (< 1)	-
Skin lesion	2 (< 1)	-	-	2 (< 1)	1 (< 1)	1 (< 1)	1 (< 1)	1 (< 1)	-	-
Skin reaction	11,029 (43)	36 (39)	7,419 (42)	3,521 (45)	4,110 (46)	6,919 (41)	8,117 (46)	2,272 (34)	483 (42)	157 (40)
Skin ulcer	157 (1)	1 (1)	101 (1)	54 (1)	74 (1)	83 (< 1)	98 (1)	48 (1)	9 (1)	2 (1)
**Vascular disorders**	**2 (< 1)**	**-**	**1 (< 1)**	**1 (< 1)**	**2 (< 1)**	**-**	**2 (< 1)**	**-**	**-**	-
Hematoma	2 (< 1)	-	1 (< 1)	1 (< 1)	2 (< 1)	-	2 (< 1)	-	-	-

AA, anaplastic astrocytoma; AE, adverse event; AO, anaplastic oligodendroglioma; MedDRA, Medical Dictionary for Regulatory Activities; ndGBM, newly diagnosed glioblastoma; rGBM, recurrent glioblastoma; TTFields, Tumor Treating Fields

^a^Includes high-grade gliomas, low-grade gliomas, and brain metastases; ^b^Commonly described as a tingling sensation below the arrays; ^c^heat below the arrays; commonly described as a warm sensation

Percentages may not total to 100%, due to rounding

TTFields therapy-related AEs were also generally similar across patient subgroups, again with the exception that the incidence of treatment-related skin reactions was slightly higher in patients with ndGBM than in those with rGBM (n=8,177 [46%] vs n=2,272 [34%] patients, respectively) (Table 3).

In total, 49 (53%) of pediatric patients reported AEs considered to be possibly related to TTFields therapy. The most common treatment-related AEs in pediatric patients were: skin reaction (n=36 [39%]), heat sensation (n=13 [14%]), and headache (n=11 [12%]). There were no device-related AEs specific to children. There were no reports of device-related agitation, insomnia, or sleep disorder in pediatric patients.

In elderly patients, 4,432 (57%) reported AEs considered to be possibly related to treatment. The most common treatment-related AEs in elderly patients were skin reaction (n=3,521 [45%]), heat sensation (n=901 [12%]), and electric sensation (n=800 [10%]). Incidence of treatment-related fall and fracture in elderly patients was 1% and <1%, respectively.

Incidence of treatment-related skin reaction was 41% (n=6,919) and 46% (n=4,110) in males and females, respectively.

Patients with ≥1 all-cause serious AE are shown by subgroup in Supplementary Table 2). Serious AEs were reported in 5,773 (22%) of patients overall, with seizure (n=1,930 [7%]), brain edema (n=637 [2%]), fall (n=622 [2%]), general physical health deterioration (n=418 [2%]), and respiratory tract infection (n=406 [2%]) being the most common. Serious AEs considered potentially related to TTFields therapy were reported by in 124 (<1%) patients (Table 4); most common were wound complication (n=86 [<1%]), skin ulcer (n=20 [<1%]), skin laceration (n=7 [<1%] beneath the arrays), and fall (n=7 [<1%]).

**Table 4:** Serious AEs potentially related to TTFields therapy in total patient cohort

MedDRA v25.1 System Organ Class / preferred term n (%)	Total (N = 25,898)
**Patients with ≥ 1 serious device-related event**	**124 (< 1)**
**General disorders and administration site conditions**	**1 (< 1)**
Gait disturbance	1 (< 1)
**Infections and infestations**	**6 (< 1)**
Abscess	3 (< 1)
Brain abscess	1 (< 1)
Cellulitis	1 (< 1)
Osteomyelitis	1 (< 1)
**Injury, poisoning and procedural complications**	**101 (< 1)**
Contusion	1 (< 1)
Fall	7 (< 1)
Fracture	6 (< 1)
Injury	1 (< 1)
Radiation injury	1 (< 1)
Skin laceration	7 (< 1)
Wound complication^a^	86 (< 1)
**Nervous system disorders**	**6 (< 1)**
Cognitive disorder	1 (< 1)
Headache	6 (< 1)
**Psychiatric disorders**	**1 (< 1)**
Anxiety	1 (< 1)
**Skin and subcutaneous tissue disorders**	**26 (< 1)**
Skin erosion	4 (< 1)
Skin reaction	2 (< 1)
Skin ulcer	20 (< 1)

AE, adverse event; MedDRA, Medical Dictionary for Regulatory Activities; TTFields, Tumor Treating Fields

^a^Includes wound dehiscence and wound infection

Only 405 (2%) of patients overall reported an AE of TTFields-related ‘QoL decreased’. In the context of these analyses, ‘QoL decreased’ refers to an unsolicited AE incidence of ‘QoL decreased’ per MedDRA version 25.1 preferred terms; QoL analysis via validated QoL assessment scale was not performed.

No new safety signals were identified in any patient subgroup, including vulnerable pediatric and elderly patients, and no systemic toxicities were reported.

## Discussion

This retrospective, global, PMS analysis represents the largest dataset of TTFields therapy-treated patients to date, including more than 25,000 patients with CNS tumors over an 11-year period, and adds to the growing body of real-world safety data for TTFields therapy. These long-term data demonstrate the tolerability of TTFields therapy in patients with CNS malignancies.

The baseline characteristics of the dataset were reflective of the real-world demographics for this patient population [6]. In line with the literature, most patients in this dataset had a diagnosis of ndGBM. [35].

Rates and types of AEs were generally comparable regardless of age, sex, or diagnosis, demonstrating feasibility of TTFields therapy across demographic subgroups, including high-risk pediatric and elderly populations.

Goldman et al. previously reported pediatric data (n=81) from the same TTFields therapy PMS dataset from 2015–2021 [34]. Overall, AEs were predominantly mild-to-moderate localized skin events; incidence of skin reactions was similar in each age group (35% in children <13 years of age and 37% in adolescents 13–17 years of age) [34]. There were no unexpected toxicities identified in the pediatric patient population and, our analysis, representing an expansion of this dataset, continues to align with these findings. Although the TTFields therapy label does not currently include use in children with GBM (it is approved in adults [≥22 years of age in the US and ≥18 years of age in all other countries]), owing to their exclusion from the pivotal EF-11 and EF-14 studies, there is a growing body of real-world data and case reports indicating feasibility, in terms of safety, in pediatric patients [34, 36, 37].

Ram et al. recently conducted a subgroup analysis of the pivotal EF-14 study in elderly patients (≥65 years of age) with ndGBM. TTFields therapy concurrent with maintenance TMZ significantly improved PFS, and TTFields therapy-related skin AEs were low-grade and manageable, with no significant increases in systemic toxicity or negative effects on patient health-related QoL [38]. There were no major differences in AEs between patients >65 years of age and the overall population. Furthermore, TTFields therapy-related falls and fractures (which could perhaps occur due to the need to carry the device) were uncommon in elderly patients; this is important in a patient population who may be more susceptible to such events.

Real-world data to date also indicate no new safety findings or added toxicities of TTFields therapy in patients with electronic implantable devices including cardiac pacemakers/defibrillators and programmable/non-programmable VP shunts [33, 39, 40].

As expected from experiences with the clinical studies, and previous real-world data, the most common TTFields therapy-related AEs were mild-to-moderate localized skin AEs beneath the arrays [4, 9, 23, 32, 41–47]. The incidence of treatment-related skin reactions was slightly higher in patients with ndGBM than with rGBM (46% vs 34%, respectively), in agreement with previous reports [4, 34, 48], and which may be secondary to cumulative toxic effects of TMZ, and more likely skin sensitization caused by radiation therapy immediately preceding TTFields therapy in patients with ndGBM. The incidence of treatment-related skin reactions in patients with astrocytoma/oligodendroglioma was 42%, providing evidence of the device safety profile in this diagnostic subset for which fewer TTFields therapy data have been published previously, compared with GBM. In patients with ‘other’ tumor types (which included low-grade gliomas, high-grade gliomas, and metastases), the incidence of treatment-related skin reactions was 40%, further highlighting consistency of the device safety profile among diagnostic groups.

Skin AEs associated with TTFields therapy are, amongst other factors, a result of the mechanical, thermal, chemical, and moisture-related stresses related to prolonged contact between the skin and the device arrays and adhesive [49, 50]. Dermatologic irritations can include contact dermatitis, hyperhidrosis, xerosis or pruritus, and more rarely, skin erosions/ulcers and infections [49]. Such AEs can be mitigated, and scalp health maintained, using prophylaxis techniques, such as careful application, removal, and repositioning of the arrays, alongside regular skin examinations [49, 51–53]. Education of patients and caregivers about such preventative strategies, together with optimization of clinical practice techniques, may therefore reduce the risk of developing skin irritations [51, 54]. Prevention and management of skin AEs may maximize TTFields therapy usage time, which has been shown to be associated with improved survival outcomes in previous studies [28, 55].

When skin irritations are reported, these are typically mild-to-moderate and can usually be managed using topical agents, such as over-the-counter steroid creams, with referral to a dermatologist required only in the case of Grade 2 or 3 AEs [49].

Importantly, and in line with observations from the pivotal GBM clinical studies, no notable TTFields therapy-related systemic AEs were detected in this long-term surveillance analysis [9, 23]. Similarly, prospective clinical studies have consistently shown that TTFields therapy given concomitantly with systemic standard of care therapies does not lead to increases in systemic toxicity versus the concomitant therapy alone, in patients with GBM [56–58]. The lack of systemic side effects can be attributed to the locoregional nature of the therapy and is a key consideration in the GBM patient population who are already heavily burdened by disease and symptomology, as well as by considerable side effects of concurrent systemic treatments.

As this analysis spans 11 years, from 2011 to 2022, the database includes patients who have used the first generation NovoTTF-100A system, and those who have used the next generation NovoTTF-200A system. The second-generation system was CE-marked in the EU in 2015 and approved in the USA by the US Food and Drug Administration in 2016. The NovoTTF-200A is smaller and lighter than its predecessor, allowing for increased convenience and manageability. The transducer arrays themselves did not change largely as part of the system update; the only difference was a change in array color from white to tan. Patient experiences with the different generations of the device were not captured in this analysis; however, survey data in patients with GBM indicate that the improved handling and portability and overall design modifications of the second-generation NovoTTF-200A system help patients comply with daily treatment duration goals and meet recommended target compliance rates required for optimal therapeutic efficiency [59].

The large cohort size is a major strength of this analysis. There are, however, limitations inherent to the retrospective study design. Analyses were not statistically powered based on the design, and therefore, comparative statements should be considered as observational only. The retrospective nature of the study design has the potential to underestimate frequency of AEs. It is possible that the duration of treatment may impact occurrence and severity of skin AEs; however, these data were not captured. Additionally, data were not collected regarding use of concomitant therapies which may have had an effect on AE incidences.

An ongoing pivotal study assessing TTFields therapy in combination with radiotherapy and TMZ in ndGBM (TRIDENT; NCT04471844), and ongoing pivotal and pilot studies in other solid tumors, including pancreatic cancer and brain metastases from lung cancer, will provide further safety data of TTFields therapy across additional patient populations.

## Conclusions

This global, PMS analysis of >25,000 patients represent the largest dataset of TTFields therapy use to date. These long-term, real-world data expand on previous real-world and clinical data, demonstrating a tolerable safety profile of TTFields therapy for patients with CNS tumors, with no new safety findings. Most AEs were localized and manageable non-serious skin events. There were no systemic adverse effects related to device use reported.

Overall, the safety profile of TTFields therapy remained consistent among patient subgroups and the total cohort, suggesting feasibility in multiple subpopulations. TTFields therapy was well tolerated across diagnostic groups, including patients with astrocytoma/oligodendroglioma and other CNS tumors. Importantly, the safety profile in pediatric and elderly patient subgroups was consistent with the overall population, with no new safety signals identified in these vulnerable populations. These data therefore provide further supportive evidence of the broad applicability of TTFields therapy.

### Supplementary Information

Below is the link to the electronic supplementary material.

Supplementary file1 (DOCX 53 KB)

## Data Availability

Analyzed, non-confidential data will be made available 3 years after the date of publication upon reasonable request from qualified researchers.
