# Supplementary material for: Global post‑marketing safety surveillance of Tumor Treating Fields (TTFields) therapy in over 25,000 patients with CNS malignancies treated between 2011–2022
Source: J Neurooncol. 2024 Jun 29;169(1):25–38. doi: 10.1007/s11060-024-04682-7 (PMC11269345; doi:10.1007/s11060-024-04682-7)
Supplement: Supplementary file 1 — Supplementary file1 (DOCX 53 KB) [file 11060_2024_4682_MOESM1_ESM.docx]

**Supplementary material**

**Supplementary Table 1**. Most common TTFields therapy-related AEs by age, sex, and diagnosis, with incidence of ≥2% in the total cohort

| **MedDRA v25.1 System Organ Class** / preferred term **n (%)** | **Total (N=25,898)** | **Age (years)** | | | **Sex** | | **Diagnosis** | | | |
| --- | --- | --- | --- | --- | --- | --- | --- | --- | --- | --- |
|  |  | **<18**  **(n=93)** | **18–65**  **(n=17,817)** | **>65**  **(n=7,808)** | **Female**  **(n=8,904)** | **Male**  **(n=16,994)** | **ndGBM**  **(n=17,587)** | **rGBM**  **(n=6,774)** | **AA/AO**  **(n=1,141)** | **Other^a^**  **(n=396)** |
| **Patients with ≥1 related AE** | **14,599 (56)** | **49 (53)** | **10,050 (56)** | **4,432 (57)** | **5,333 (60)** | **9,266 (55)** | **10,491 (60)** | **3,249 (48)** | **635 (56)** | **224 (57)** |
| **General disorders and administration site conditions** | **7,356 (28)** | **22 (24)** | **5,250 (29)** | **2,056 (26)** | **2,797 (31)** | **4,559 (27)** | **5,232 (30)** | **1,675 (25)** | **337 (30)** | **112 (28)** |
| Discomfort | 609 (2) | 2 (2) | 414 (2) | 192 (2) | 239 (3) | 370 (2) | 458 (3) | 113 (2) | 29 (3) | 9 (2) |
| Electric Sensation^a^ | 3,557 (14) | 9 (10) | 2,734 (15) | 800 (10) | 1,402 (16) | 2155 (13) | 2,576 (15) | 758 (11) | 167 (15) | 56 (14) |
| Fatigue / Malaise | 1,337 (5) | 5 (5) | 871 (5) | 455 (6) | 461 (5) | 876 (5) | 982 (6) | 287 (4) | 52 (5) | 16 (4) |
| Heat Sensation^b^ | 3,083 (12) | 13 (14) | 2,157 (12) | 901 (12) | 1,156 (13) | 1,927 (11) | 2,152 (12) | 726 (11) | 152 (13) | 53 (13) |
| Pain | 1,227 (5) | 3 (3) | 852 (5) | 371 (5) | 566 (6) | 661 (4) | 913 (5) | 251 (4) | 51 (4) | 12 (3) |
| **Injury, poisoning and procedural complications** | **496 (2)** | **-** | **323 (2)** | **167 (2)** | **234 (3)** | **262 (2)** | **365 (2)** | **109 (2)** | **16 (1)** | **6 (2)** |
| **Investigations** | **405 (2)** | **-** | **280 (2)** | **124 (2)** | **149 (2)** | **256 (2)** | **328 (2)** | **59 (1)** | **13 (1)** | **5 (1)** |
| Quality of Life Decreased | 405 (2) | - | 280 (2) | 124 (2) | 149 (2) | 256 (2) | 328 (2) | 59 (1) | 13 (1) | 5 (1) |
| **Nervous system disorders** | **2,251 (9)** | **11 (12)** | **1,708 (10)** | **526 (7)** | **891 (10)** | **1,360 (8)** | **1,544 (9)** | **572 (8)** | **94 (8)** | **41 (10)** |
| Headache | 2,144 (8) | 11 (12) | 1,639 (9) | 488 (6) | 845 (9) | 1,299 (8) | 1,463 (8) | 550 (8) | 91 (8) | 40 (10) |
| **Skin and subcutaneous tissue disorders** | **11,193 (43)** | **36 (39)** | **7,550 (42)** | **3,554 (46)** | **4,147 (47)** | **7,046 (41)** | **8,228 (47)** | **2,314 (34)** | **493 (43)** | **158 (40)** |
| Hyperhidrosis | 394 (2) | 1 (1) | 311 (2) | 81 (1) | 95 (1) | 299 (2) | 296 (2) | 74 (1) | 19 (2) | 5 (1) |
| Skin Reaction | 11,029 (43) | 36 (39) | 7,419 (42) | 3,521 (45) | 4,110 (46) | 6,919 (41) | 8,117 (46) | 2,272 (34) | 483 (42) | 157 (40) |

AA, anaplastic astrocytoma; AE, adverse event; AO, anaplastic oligodendroglioma; MedDRA, Medical Dictionary for Regulatory Activities; ndGBM, newly diagnosed glioblastoma; rGBM, recurrent glioblastoma; TTFields, Tumor Treating Fields. ^a^Includes high-grade gliomas, low-grade gliomas, and brain metastases; ^b^Electric (tingling) sensation; ^c^heat (warmth) sensation.

**Supplementary Table 2. Patients with ≥1 all-cause serious AE, by age, sex, and diagnosis**

| **MedDRA v25.1 System Organ Class /** preferred term **n (%)** | **Total (N=25,898)** | **Age (years)** | | | **Sex** | | **Diagnosis** | | | | |
| --- | --- | --- | --- | --- | --- | --- | --- | --- | --- | --- | --- |
|  |  | **<18**  **(n=93)** | **18–65**  **(n=17,817)** | **>65**  **(n=7,808)** | **Female**  **(n=8,904)** | **Male**  **(n=16,994)** | **ndGBM**  **(n=17,587)** | | **rGBM**  **(n=6,774)** | **AA/AO**  **(n=1,141)** | **Other^a^**  **(n=396)** |
|  | 5,773 (22) | 13 (14) | 3,756 (21) | 1,975 (25) | 2,033 (23) | 3,740 (22) | 4,021 (23) | 1,492 (22) | | 180 (16) | 80 (20) |

AA, anaplastic astrocytoma; AE, adverse event; AO, anaplastic oligodendroglioma; MedDRA, Medical Dictionary for Regulatory Activities; ndGBM, newly diagnosed glioblastoma; rGBM, recurrent glioblastoma; TTFields, Tumor Treating Fields.
^a^Includes high-grade gliomas, low-grade gliomas and brain metastases.
